# Inhibiting breast cancer by targeting the thromboxane A_2_ pathway

**DOI:** 10.1038/s41698-017-0011-4

**Published:** 2017-04-03

**Authors:** Haitao Li, Mee-Hyun Lee, Kangdong Liu, Ting Wang, Mengqiu Song, Yaping Han, Ke Yao, Hua Xie, Feng Zhu, Michael Grossmann, Margot P. Cleary, Wei Chen, Ann M. Bode, Zigang Dong

**Affiliations:** 1 The Hormel Institute, University of Minnesota, Austin, MN USA; 2 The China-US (Henan) Hormel Cancer Institute, Zhengzhou, Henan China; 3 School of Food Science and Technology, Jiangnan University, Wuxi, Jiangsu China

## Abstract

Targeting the estrogen receptor as a strategy has been the gold standard for breast cancer chemoprevention or breast cancer recurrence, but its benefit is limited to estrogen receptor-positive tumors. Cyclooxygenases have been implicated in mammary tumorigenesis. We sought to identify the key prostaglandin responsible for the pro-neoplastic effect of cyclooxygenases and develop prostaglandin-targeted strategies for breast cancer chemoprevention or therapy. Immunohistochemical analysis revealed that either thromboxane A_2_ synthase 1 or the thromboxane A_2_ receptor is highly expressed in human breast tumors as well as premalignant lesions, but not in normal mammary tissues. Clinically, the thromboxane A_2_ pathway might be associated with HER2-positive and axillary lymph node metastasis in human breast cancer. We found that the thromboxane A_2_ pathway was required for breast cancer cell growth, anchorage-independent growth and invasion capabilities. Importantly, we discovered that switching off thromboxane A_2_ biosynthesis effectively suppressed either MMTV-HER2-driven mammary tumorigenesis or breast cancer metastasis in preclinical animal models. Taken together, this study established a critical pathophysiological role of the thromboxane A_2_ pathway in breast cancer, and provided a rationale for introducing a strategy targeting thromboxane A_2_ for breast cancer chemoprevention and therapy.

## Breast cancer: Pathway target identified

The identification of a signaling pathway connected to the progression of breast cancer could prove a valuable therapeutic target. The breast cancer treatment tamoxifen, although successful, is limited to certain tumor types, and so the search is on to pinpoint molecular targets that are ubiquitous across breast cancers. Zigang Dong at the University of Minnesota, US, and co-workers identified that the thromboxane A_2_ (TXA_2_) pathway is highly-expressed in human breast tumors and premalignant lesions. Further examination showed that TXA_2_ is crucial for tumor cell growth and metastasis. When the researchers knocked-out TXA_2_ production in mouse models, this suppressed both the formation and spread of tumors. The team advise caution because TXA_2_ is also required for wound healing and other key processes, although short-term bursts of pathway suppression may be an option for breast cancer therapy.

## Introduction

Breast cancer represents the most common cancer in women worldwide.^1^ Fortunately, during breast carcinogenesis, the transition from normal milk ducts to invasive ductal carcinoma is a protracted event that offers opportunities for preventive intervention.^2^ For example, targeting the estrogen receptor (ER) has been the gold standard for breast cancer chemoprevention, and tamoxifen was the first chemopreventive agent approved by the Food and Drug Administration for both primary and secondary breast cancer prevention.^3^ However, the benefit of tamoxifen is mainly limited to ER-positive tumors and, unfortunately, tamoxifen and/or other selective estrogen response modifiers might increase the risk of endometrial cancer and cardiovascular events.^4^ Therefore, identifying novel preventive agents is thought to be an essential step forward, which is largely dependent upon the discovery of precise molecular targets for breast cancer chemoprevention or therapy to prevent recurrence after surgery.

Cyclooxygenases (COXs) have been recently implicated in the etiology of breast cancers and are known to exert their biological function through prostaglandins (PGs).^5, 6^ Although COXs are still among the most promising molecular targets for chemoprevention, side effects have greatly dampened enthusiasm for their long-term inhibition.^7–9^ The COX enzyme exists in two isoforms, COX-1 and COX-2. COX-1 was generally considered to be a housekeeping gene responsible for basal PG biosynthesis under normal physiological conditions, whereas COX-2 is an immediate-early response gene that is induced under various pathophysiologies such as inflammation and tumorigenesis. Selective COX-1 inhibition might cause gastrointestinal toxicity, whereas selective COX-2 inhibition might increase the risk of cardiovascular events. To overcome these issues, one theoretical approach is to identify the key PG responsible for the pro-neoplastic effect of COXs in breast cancer and then to develop strategies to inhibit that PG selectively. Among the five major bioactive PGs, thromboxanes A_2_ (TXA_2_) drew our attention. Aspirin intake has been associated with a lower risk of breast cancer incidence and mortality in epidemiological studies^10–14^ and pharmacokinetic data strongly suggest that aspirin might target COX-1 as well as its downstream pro-thrombotic TXA_2_ biosynthesis.^15, 16^ Recently, thromboxane A_2_ synthase 1 (TBXAS1) polymorphism was reported to be associated with breast cancer susceptibility.^17^ These findings prompted us to examine whether the TXA_2_ pathway functionally mediates breast tumorigenesis, and to clarify the underlying mechanism(s) of action.

## Results

### The TXA_2_ pathway is constitutively activated during human breast cancer development

**Fig. 1:**
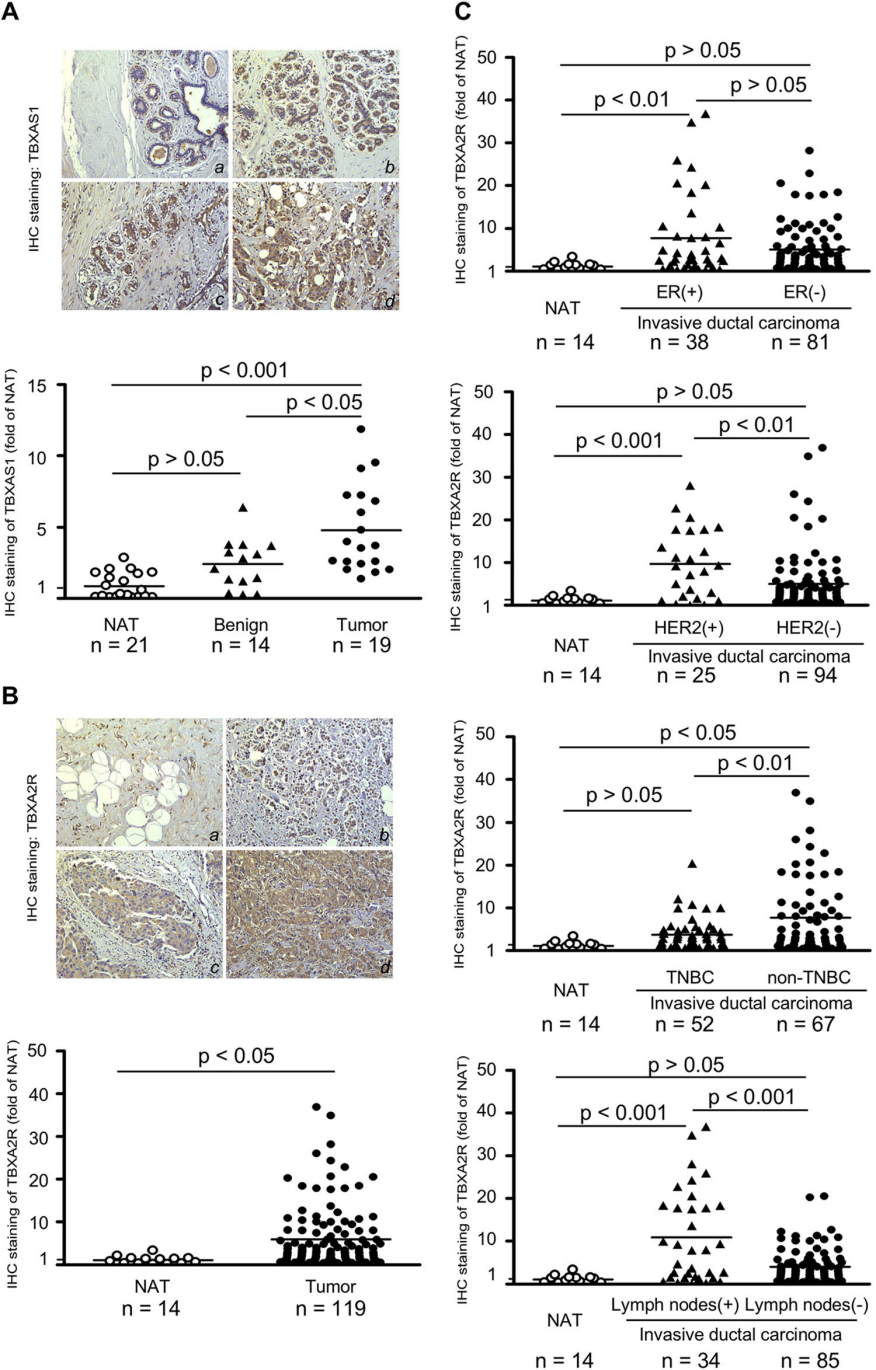
Pathophysiological role of the TXA_2_ pathway in human breast cancer. **a** Immuno-histochemical staining of TBXAS1 in human breast cancer tissues. (a) normal adjacent breast tissues; (b) adenosis of breast; (c) atypical hyperplasia of duct epithelium; (d) infiltrating duct carcinoma. Original magnification: 200×. **b** Immunohistochemical staining of TBXA2R in human breast cancer. (a) normal adjacent breast tissues; (b–d) infiltrating duct carcinoma. Original magnification: 200×. **c** Clinical relevance of TBXA2R in human breast cancer. For **a**, **b**, **c**, the *graphs* show the staining of TBXAS1 (**a**) or TBXA2R (**b**, **c**) and the *horizontal line* indicates the mean value. The *asterisks* indicate a significant difference compared with the NAT (normal adjacent tissue) group (*p < 0.05; **p < 0.01; ***p < 0.001)

To clarify the importance of the TXA_2_ pathway in breast cancer, we first examined the expression of TBXAS1, a rate-limiting enzyme coupled with COXs in the synthesis of TXA_2_.^6, 18^ Our immunohistochemical data confirmed that TBXAS1 expression in breast tumors was 4.6-fold higher than observed in normal adjacent tissues (NAT) (Fig. 1a). Interestingly, TBXAS1 expression was also more likely to increase in breast precancerous lesions such as breast adenosis, epithelium hyperplasia and atypical ductal hyperplasia. In general, the staining pattern was cytoplasmic and granular. Within the same tissue sections, TBXAS1 staining was localized mainly in tumor cells instead of stromal cells. TBXAS1 staining in those normal-appearing mammary ductular epithelia was often focal and of reduced in intensity relative to neoplastic epithelia. Because TXA_2_ is known to function through the activation of the thromboxane A_2_ receptor (TBXA2R), we further investigated TBXA2R expression in breast cancer and observed that the protein levels of TBXA2R in breast tumors were 5.9-fold higher than NAT (Fig. 1b). Regarding its potential clinical relevance, TBXA2R might be associated with ER, HER2, and axillary lymph node metastases (Fig. 1c).

### The TXA_2_ pathway is required for tumorigenic properties of breast cancer cells

**Fig. 2:**
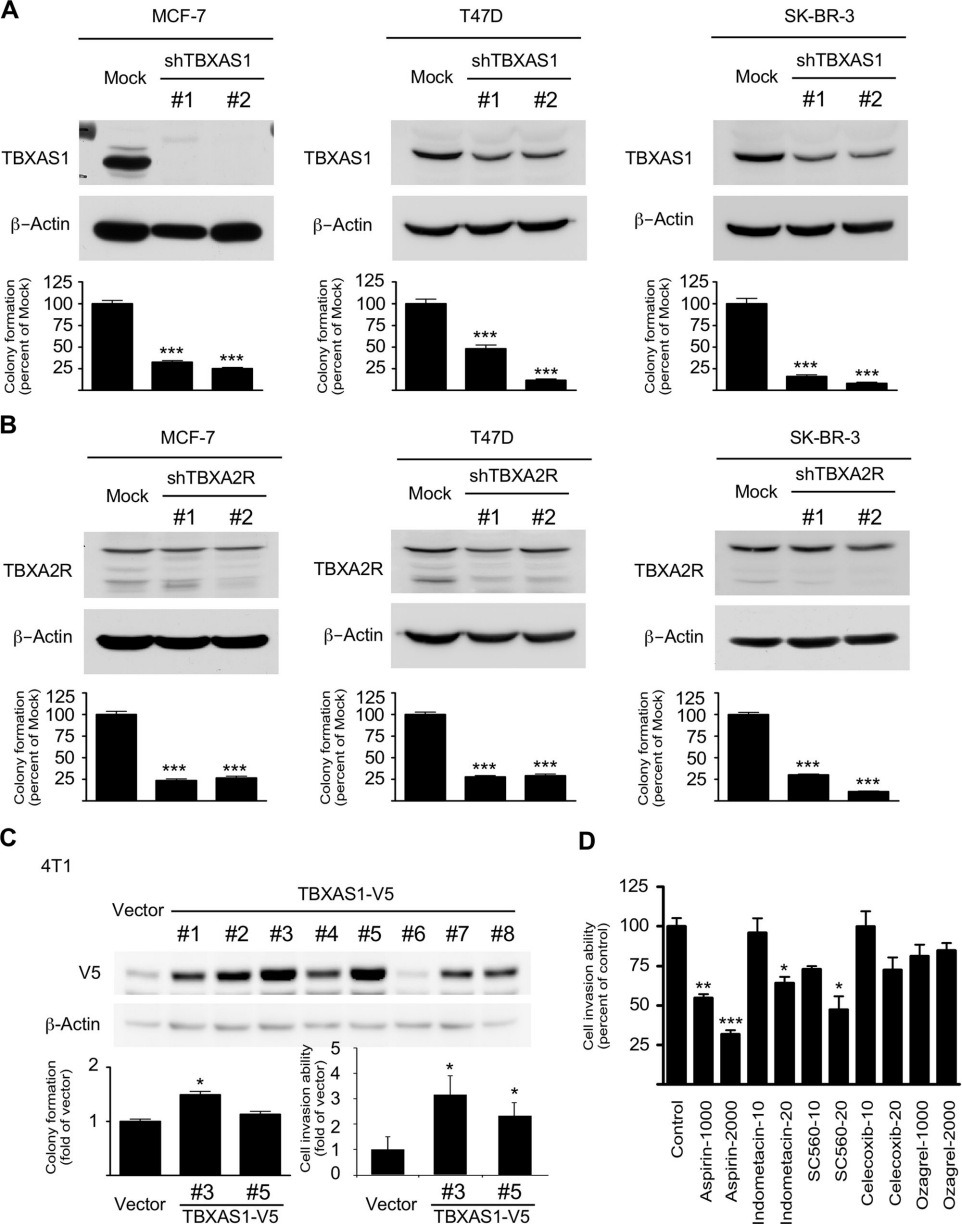
The TXA_2_ pathway is required for maintenance of malignant characteristics of MCF-7, T47D, and SK-BR-3 breast cancer cells. **a** Knockdown of TBXAS1 inhibits anchorage-independent growth of human breast cancer cells. **b** Knockdown of TBXA2R inhibits anchorage-independent growth of human breast cancer cells. Knockdown of TBXAS1 or TBXA2R in breast cancer cells was analyzed by Western blot. Anchorage-independent cell growth was evaluated by colony formation in soft-agar. *Data* are presented as mean values ± S.E.M. from three independent experiments. The *asterisks* (***) indicate a significant (*p* < 0.001) decrease compared to the Mock group. **c** Effect of ectopic expression of TBXAS1 on anchorage-independent cell growth and invasion. At 50–60% confluence, 4T1 cells were transiently transfected with either an empty vector (pcDNA3.1) or a TBXAS1 plasmid (pcDNA3.1-V5-TBXAS1). After 24 h, G418 (1200 ng/mL) was added for selection of stable subclones. After 3 week, the stable clones obtained were verified by Western blot. Anchorage-independent cell growth was evaluated by colony formation in soft-agar. Cell invasion was determined by transwell assay. Data are presented as mean values ± S.E.M. from three independent experiments. The *asterisks* indicate a significant difference compared with vector group (**p < 0.01; ***p < 0.001). **d** Effect of NSAIDs on cell invasion. 4T1 cells were plated in a Matrigel-coated upper chamber with or without NSAIDs in the medium. After incubation for 24 h, non-migrated cells on the upper surface of the filter were wiped out, and cells on the lower surface of the membrane were stained with crystal violet and counted under a light microscope. The *asterisks* indicate a significant difference compared with the control group (*p < 0.05; **p < 0.01; ***p < 0.001)

During the immunohistochemical study, we noticed that positive regions of TBXAS1 or TBXA2R immunostaining were mainly in mammary ducts from where primary breast cancer is believed to arise (Fig. 1a). Accordingly, we hypothesized that the TXA_2_ pathway might be directly associated with the tumorigenic properties of breast cancer cells. Anchorage-independent growth ability is a key characteristic of the transformed cell phenotype.^19^ We found that knockdown of TBXAS1 or TBXA2R greatly impaired the anchorage-independent growth capability in MCF-7, T47D, and SK-BR-3 human breast cancer cell lines, evidenced by fewer colonies forming in soft agar compared with Mock (Fig. 2a, b). Moreover, knockdown of TBXAS1 or TXA_2_R lowered the rate of cancer cell proliferation (Supplementary Fig. 1a, b). To further characterize the role of the TXA_2_ pathway in breast cancer, we successfully established TBXAS1-overexpressing stable sub-clones from murine mammary carcinoma 4T1cells, which expressed very low levels of endogenous TBXAS1. Moreover, 4T1 tumor cell invasion ability was increased by overexpression of TBXAS1 (Fig. 2c), whereas it was ameliorated by aspirin, indomethacin, or SC560 treatment (Fig. 2d). Notably, all of the TXA_2_ modulators tested, except indomethacin and celecoxib, also down-regulated TBXAS1 expression. However, the interpretation of this phenomenon is still unclear (Supplementary Fig. 2).

### Chemoprevention of HER2-positive breast cancer by targeting the TXA_2_ pathway

**Fig. 3:**
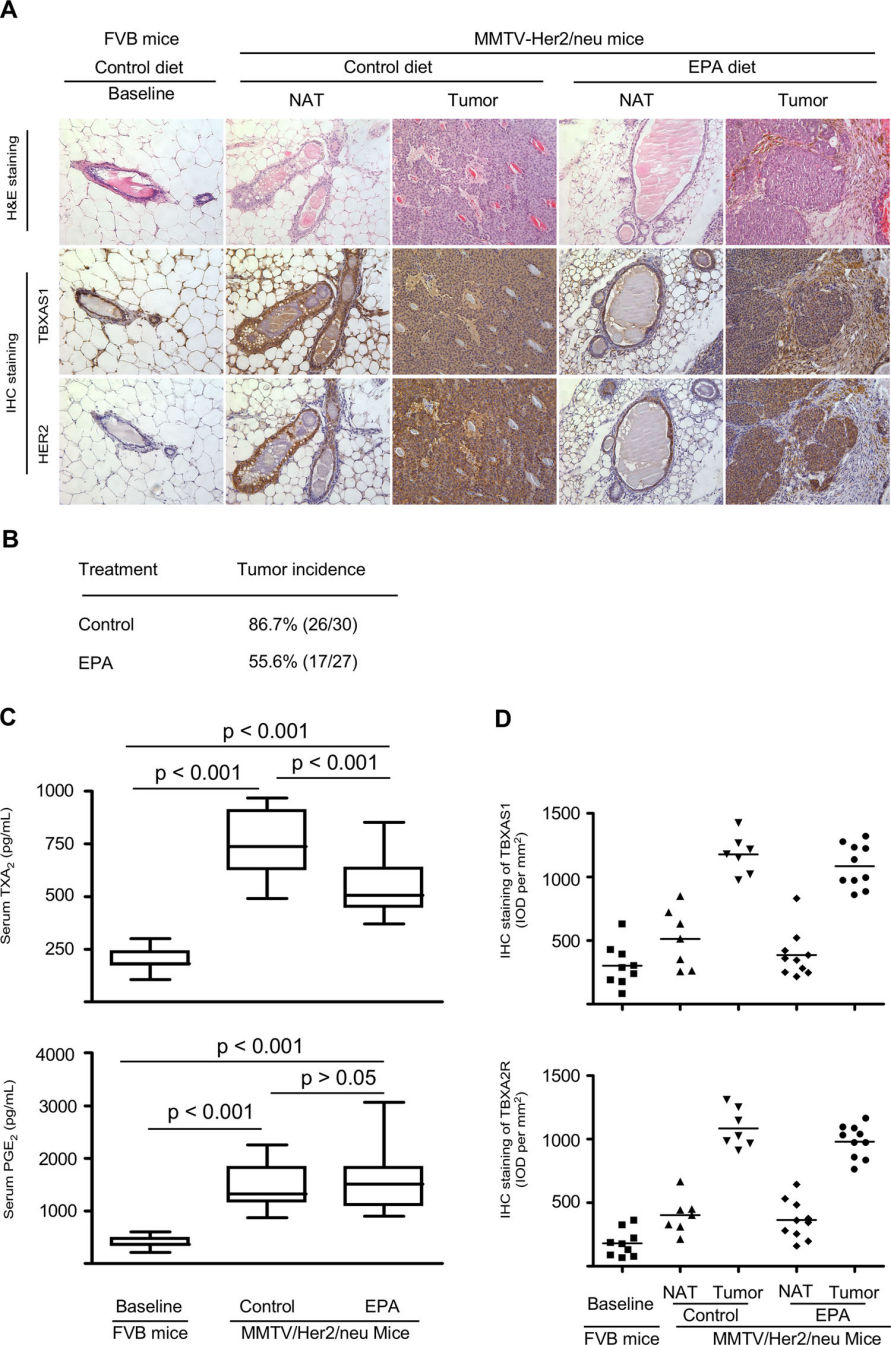
Chemoprevention of HER2-positive breast cancer by targeting theTXA_2_ pathway. **a** TBXAS1 is up-regulated in HER2-driven mammary tumorigenesis in an MMTV-Her2/neu transgenic mouse mammary tumor model. Immunohistochemical staining of TBXAS1 in mouse mammary tumors or normal mammary tissues. Original magnification: 200×. **b** Switching off TXA_2_ biosynthesis attenuates HER2-driven breast tumorigenesis. MMTV-HER2/neu transgenic mice were treated with EPA for a total of 60 weeks. **c** Effects of EPA intake on circulating TXA_2_ and PGE_2_ levels. **d** Effects of EPA intake on TBXAS1 or TBXA2R in the MMTV-neu mammary glands and tumors. Because the FVB background of MMTV-Her2/neu transgenic mice, matched non-transgenic FVB mice were used as a baseline to minimize the genetic differences. Production of serum PGs was measured by ELISA as described in “Materials and Methods”. Data are presented as mean values ± S.E.M. The *asterisks* indicate a significant difference among groups (*p < 0.05, ***p < 0.001)

We next questioned whether the TXA_2_ pathway could serve as a potential target for breast cancer chemoprevention. By using an MMTV-Her2/neu transgenic mouse mammary tumor model, we established that TBXAS1 and HER2 were co-overexpressed and co-localized in the mammary ducts (Fig. 3a). Eicosapentaenoic acid (EPA), an ingredient of fish oil, has a similar chemical structure as arachidonic acid (AA) and thus might act as a competitive inhibitor of TBXAS1, being converted to TXA_3_ instead of TXA_2_.^18^ EPA intake significantly lowered breast cancer incidence by 31% (*p* < 0.05) and the circulating TXA_2_ levels by 27% (*p* < 0.001), respectively (Fig. 3b, c). EPA also slightly reduced TBXAS1 expression both in the MMTV-neu mammary gland and tumor, but the difference was not statistically significant (Fig. 3d, Supplementary Fig. 3). EPA was well tolerated in mice and no obvious systemic toxicity was observed during the entire period of drug treatment as indicated by body weight, general appearance, and organ histology. Notably, EPA intake greatly lowered circulating TXA_2_ levels, but had little effect on PGE_2_ levels, indicating that interfering with TXA_2_ biosynthesis alone might be sufficient to reduce the risk of HER2-driven breast tumorigenesis.

### Attenuation of breast cancer metastasis by targeting the TXA_2_ pathway

**Fig. 4:**
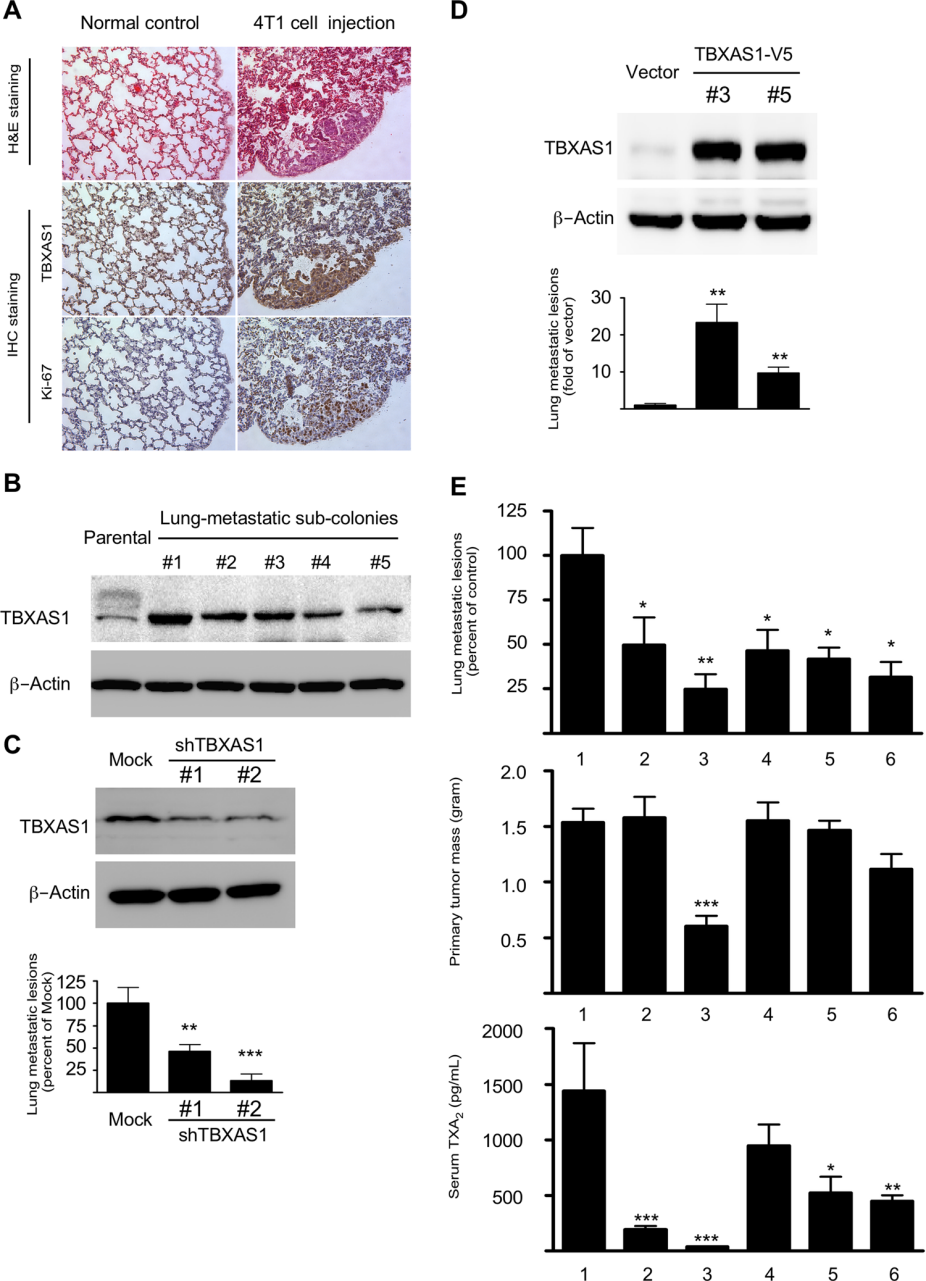
Breast cancer metastasis is attenuated by targeting the TXA_2_ pathway. **a** Murine 4T1 tumor cells spontaneously metastasize from the primary tumor (mammary gland) to the lung. Immunohistochemical staining showed that metastatic foci in lung strongly express TBXAS1. **b** Compared with parental cells, 4T1sub-line cells isolated from lung metastatic foci generally express much higher levels of TBXAS1. **c** Knockdown of TBXAS1 decreases the ability of 4T1 cells to metastasize to the lung. Data are presented as mean values ± S.E.M. (*n* = 6). The *asterisks* indicate a significant difference compared with Mock control (**p < 0.01; ***p < 0.001). **d** Ectopic expression of TBXAS1 increases the ability of 4T1 cells to metastasize to the lung. Data are presented as mean values ± S.E.M. (*n* = 6). The *asterisks* indicate a significant difference compared with vector control (**p < 0.01). **e** Pharmacological blockage of TXA_2_ biosynthesis suppresses breast cancer metastasis to the lung. Drug treatment: 1, vehicle; 2, aspirin (100 mg/kg); 3, indomethacin (1 mg/kg); 4, celecoxib (10 mg/kg); 5, ozagrel sodium (100 mg/kg), 6, fish oil (500 mg/kg). Data are presented as mean values ± S.E.M. (*n* = 10). The *asterisks* indicate a significant difference compared with vehicle control (*p < 0.05, **p < 0.01, ***p < 0.001)

Despite decades of advances in therapeutic regimens, metastasis remains the leading cause of breast cancer-related death.^20, 21^ We then addressed whether the TXA_2_ pathway functionally mediated breast cancer metastasis. Murine mammary carcinoma 4T1cells are highly tumorigenic and can spontaneously metastasize from the primary tumor (i.e., mammary gland) to distant sites such as lung, brain, and bone.^22^ We observed that metastatic foci in lung were strongly stained with TBXAS1 (Fig. 4a). Compared with parental cells, 4T1 sub-line cells isolated from lung metastatic foci generally expressed higher levels of TBXAS1 (Fig. 4b). Importantly, the metastatic capacity of 4T1cells was impaired by TBXAS1 knockdown (Fig. 4c), whereas metastatic capacity was enhanced by TBXAS1 overexpression (Fig. 4d). Remarkably, nearly a 200-fold difference was observed between over-expression and knockdown of TBXAS1. To evaluate the clinical potential of a TXA_2_-targeting strategy, we further examined the efficacy of TXA_2_ pathway modulators on breast cancer metastasis to lung (Fig. 4e). Drugs affecting TXA_2_ action can mainly be classified into three categories: COX inhibitors, TBXAS1 inhibitors, and TBXA2R antagonists. All drugs were well tolerated in mice and no obvious systemic toxicity was observed during the entire period of study. Among the drugs tested, indomethacin was the most promising because it dramatically lowered circulating TXA_2_ levels and suppressed both experimental metastasis and primary tumor growth. In contrast, aspirin, celecoxib, ozagrel, and fish oil affected breast cancer metastatic risk, but not primary tumor growth. Consistent with findings shown in Supplementary Fig. 2, indomethacin had no effect on TBXAS1 staining in the lung metastatic lesions. However, determining TBXAS1 staining might be an oversimplification and should be interpreted carefully. This is because TBXAS1 functions in TXA_2_ biosynthesis, which depends upon increased protein expression, catalytic activity, or both.

## Discussion

Although a large body of evidence indicates that PGE_2_ might be the predominant PG in cancer, the concept that PGE_2_ is the only PG involved in carcinogenesis has long been challenged. Notably, PGD_2_ functions as a pro-resolution mediator in ulcerative colitis,^23^ and PGI_2_ is the major PG in ovarian epithelial cancer.^24^ In addition to its pivotal role in platelet aggregation, a growing body of evidence indicates that the TXA_2_ pathway might be involved in the etiology of breast cancer. For example, among the five major PGs, TXA_2_ was the only AA metabolite that correlated with clinical variables such as tumor size, lymph node-positive and distant metastasis in human breast cancer.^25^ TBXAS1 polymorphism was recently reported to be associated with breast cancer susceptibility.^17^ In this study, we demonstrated that TBXAS1 expression was up-regulated not only in breast tumors but also in various precancerous lesions (Fig. 1a). The positive regions of TBXAS1 immunostaining were mammary ducts from where primary breast cancer is believed to arise. Importantly, switching off TXA_2_ biosynthesis greatly attenuated HER2-driven mammary tumorigenesis. Although in this study, we found that either ER or HER2 cases have significant levels of TBXA2R, the observation above should be interpreted carefully because the observation of ER and HER2 double positive tumors is rather low in the clinic. Furthermore, a portion of HER2 or ER negative cases showed stronger TBXA2R immunostaining compared to HER2 or ER positive cases. Most likely, TBXA2R up-regulation might be implicated in modulating angiogenesis during chronic inflammation and tumor growth and thus ubiquitous in human breast cancer. Taken together, our findings in this study indicated that lowering circulating TXA_2_ levels or interfering with the TXA_2_ pathway might be a promising strategy for breast cancer chemoprevention, either in ER or HER2 positive tumors.

Metastasis is frequently a final and fatal step in breast tumorigenesis. Lung, liver, bone, and brain are well-known frequent sites of breast cancer metastasis. Interestingly, most of those distant organs in breast cancer metastasis highly express TBXAS1.^21^ Aspirin intake after diagnosis of breast cancer was associated with a lower risk of distant recurrence and overall mortality,^10, 12^ whereas pharmacokinetic studies revealed that aspirin might exert its anticancer activity by lowering TXA_2_ biosynthesis.^15, 16^ In the present study, we provided compelling evidence to support the critical role of TXA_2_ pathway in breast cancer metastasis. Although this might partly explain why aspirin intake apparently benefited breast cancer patients, whether this is a primary association remains unclear. Aspirin and indomethacin lowered circulating TXA_2_ to a similar level, but differences between their respective activities on breast cancer metastasis to lung pointed to alternate mechanisms at play. On the basis of these observations, targeting the TXA_2_ pathway could represent a novel adjuvant therapeutic strategy against breast cancer metastasis.

Although our present findings are promising, several issues still need to be addressed. For example, our data were mainly based on animal studies, and thus the first and most important question is whether animal models accurately predict the efficacy of a TXA_2_-targeting strategy in human trials. To verify whether the introduction of TXA_2_-targeting strategies would better serve the interest of high-risk breast cancer patients, clinical studies need to be performed. Another question is how to translate our basic research findings into clinical use. Because the TXA_2_ pathway plays pivotal roles in platelet aggregation and wound repair, persistent intensive perturbation of the TXA_2_ pathway might not be an ideal chemopreventive strategy, and thus normalization of the TXA_2_ pathway had better be applied judiciously at discrete stages as a short-term intermittent therapy. Currently, very few specific TBXAS1 inhibitors and TBXA2R antagonists as pharmacological tools are available to examine TXA_2_-mediated events. To this end, the optimal drug and dose, as well as treatment regimen, for the clinical utility of TXA_2_-targeting strategies in breast cancer needs to be more thoroughly studied and defined. The third question is the molecular mechanism underlying TXA_2_-driven mammary tumorigenesis remains largely unclear. For example, although RNA interference knockdown of TBXAS1 suppressed the anchorage-independent growth, the ectopic expression of TBXAS1 didn’t or only slightly increased colony formation (Fig. 2c), a finding indicated that TBXAS1 itself alone might not be oncogenic. Besides, we observed that both TBXAS1 and microsomal prostaglandin E synthase-1 (mPGES1) were co-overexpressed and co-localized in the mammary ducts (Supplementary Fig. 3), but whether they cooperate with each other during mammary tumorigenesis, remains unclear. To gain a deeper insight into the role of the TXA_2_ pathway in breast cancer, further studies examining susceptibility to mammary tumorigenesis in mice with targeted deletions in specific PG synthases and/or receptors are greatly needed.

In summary, this study established for the first time that the TXA_2_ pathway functionally mediates breast cancer progression, and laid the groundwork for precision chemoprevention or therapy of breast cancer by targeting the TXA_2_ pathway.

## Materials and methods

### Reagents

Primary antibodies against human mPGES1, TBXAS1 and the TBXA2R were obtained from Cayman Chemical Company (Ann Arbor, MI). All other primary antibodies were purchased from Cell Signaling Technology (Beverly, MA). Human breast cancer tissue microarray slides (BC08023 and BR1503b) were purchased from US Biomax Inc. (Rockville, MD). Full-length cDNA for human *Tbxas1* was obtained from Addgene Inc. (Cambridge, MA). The 29-mer small hairpin RNA constructs against human *TBXAS1* and *TBXA2R* were purchased from Open Biosystems, Inc. (Huntsville, AL). All chemicals were purchased from Sigma-Aldrich (St Louis, MO) unless otherwise specified.

### Cell culture and transfection

All cell lines used in this study were obtained from the American Type Culture Collection (ATCC, Manassas, VA) and maintained following ATCC instructions. Cells were cytogenetically tested and authenticated before being frozen. Each vial of frozen cells was thawed and maintained for a maximum of 20 passages. For transfection, the jetPEI transfection reagent (Qbiogene, Inc., Montreal, Quebec, Canada) was used following the manufacturer’s instructions. For stable transfection experiments, cells were first transiently transfected with either empty vector (pcDNA3.1) or effector plasmid (pcDNA3.1-*Tbxas1*). After 24 h, G418 was added for stable clone selection. After 3 week, the individual clones obtained were ring-isolated and expanded in culture medium in the presence of G418 (1200 μg/mL). Expression of the protein of interest was verified by Western blot analysis.

### Prostaglandin determination

The measurement of PGs was performed using enzyme immunoassay kits from Cayman Chemical Company (Ann Arbor, MI) following the manufacturer’s instructions. In brief, cells (6 × 10^5^) were plated in each well of a 6-wellplate. When cells reached 80% confluence, 1 mL fresh medium was added and cells were further incubated for 24 h. Supernatant fractions were collected for PG measurement. Because PGD_2_, PGF_2α_, PGI_2_, and TXA_2_ are unstable in vivo, we measured each corresponding primary metabolite, respectively, as follows: 11-beta-PGF_2α_, 13, 14-dihydro-15-keto-PGF_2α_, 6-keto-PGF_1α_, and TXB_2_.

### Anchorage-independent cell growth assay

In each well of a 6-well plate, cells (8 × 10^3^) were suspended in Basal Medium Eagle (BME) medium (1 mL with 10% FBS and 0.33% agar) and plated over a layer of solidified BME (3 mL with 10% FBS and 0.5% agar). The cultures were incubated in a 37 °C, 5% CO_2_ incubator for 14 days and colonies in soft agar were counted under a microscope equipped with the Image-Pro Plus software program (Media Cybernetics, Bethesda, MD).

### Cell invasion assay

Cell invasion was assayed in a Matrigel invasion chamber following the manufacturer’s instructions. Briefly, 1 × 10^4^ cells were plated in the Matrigel-coated upper chamber of a Corning Costar chamber (Corning, USA). After incubation for 24 h, non-migrated cells on the upper surface of the filter were wiped out, and cells on the lower surface of the membrane were stained with crystal violet and counted under a light microscope in at least five different fields (original magnification, ×200).

### Western blot analysis

Protein samples (20 µg) were resolved by SDS-PAGE and transferred to Hybond C nitrocellulose membranes (Amersham Corporation, Arlington Heights, IL). After blocking, the membranes were probed with primary antibodies (1:1000) overnight at 4 °C. The targeted protein bands were visualized using an enhanced chemiluminescence reagent (Amersham Corporation) after hybridization with a secondary antibody conjugated with horseradish peroxidase.

### Mouse mammary tumor model

All animal care and experimental procedures were conducted following the guidelines established by the University of Minnesota Institutional Animal Care and Use Committee. All mice were maintained in The Hormel Institute Animal Facility and kept in an air-conditioned room with controlled temperature (22 ± 1 °C), humidity (65–70%), and day/night cycle (12 h light, 12 h dark). The MMTV-Her2/neu transgenic mouse mammary tumor model was adopted for breast cancer chemoprevention study.^26^ In brief, mice were fed a control diet (AIN-93M diet containing 4% soy oil) or a 5, 8, 11, 14, 17-EPA diet (modified AIN-93M diet in which soy oil was substituted with EPA) for a total of 60 weeks. Mice were weighed and examined weekly for mammary tumor onset. After euthanasia, primary mammary tumors were removed and fixed in 10% buffered formalin (pH 7.4) for 24 h for further histopathological assessment and immunohistochemical analysis. Blood was collected by orbital bleed using a syringe containing sodium citrate. Blood samples were then centrifuged at 2000×*g* for 15 min, and the resulting supernatant fraction was designated as plasma for PG measurement. To minimize genetic differences, matched non-transgenic FVB mice were used as a baseline for MMTV-Her2/neu transgenic mice that were FVB background.

The mouse 4T1 breast tumor model was adopted for a breast cancer metastasis study as previously described.^22, 27^ 4T1 mouse mammary carcinoma cells (2 × 10^5^ cells in 100 μL PBS) were injected using a 29-gage needle into the fourth mammary fat pad in 8-week-old female BALB/c mice. Aspirin (100 mg/kg), indomethacin (1 mg/kg), celecoxib (10 mg/kg) and one commercially available fish oil product (500 mg/kg) or vehicle [5%, v/v, dimethyl sulphoxide in olive oil] was administered using an intragastric tube at 0.1 mL per 10 g every other day throughout the experiment. Ozagrel sodium (100 mg/kg) was administered by subcutaneous (s.c.) injection. After 4 weeks, mice were euthanatized and primary tumors and lungs were collected. After gross examination, half of the lung was fixed for histopathological assessment and immunohistochemical analysis and the other half was used to harvest metastasizing 4T1 cells for quantification of distant-site metastases. In brief, the lungs were minced and digested with 1 mg/mL collagenase at 37 °C for 2 h. The lung tissue mixtures obtained were then dispersed with an 18-gage needle, washed, serially diluted, and plated in 6-well-plates with culture medium containing 6-thioguanine (60 μM). The plates were placed in a 37 °C 5% CO_2_ tissue culture incubator for 14 days. Metastatic colonies in lung were counted after crystal violet staining.

### Histology and immunohistochemistry

For histology, fixed tissues were embedded in paraffin, sectioned at 5 μm, and stained with hematoxylin and eosin according to standard protocols. Immunohistochemistry staining for TBXAS1 (#160715, Cayman Chemical Company; dilution 1:50), TBXA2R (#10004452, Cayman Chemical Company; dilution 1:50), mPGES1 (#160140, Cayman Chemical Company; dilution 1:50) and HER2 (HER2, #2242, Cell Signaling Technology; dilution 1:200) was performed using an ABC complex kit (PK-6100, Vector Laboratories, Burlingame, CA) following the manufacturer’s instructions. Sections were counterstained with Harris’s hematoxylin. Immunohistochemistry staining intensity was quantified by calculating the integrated optical density (IOD per mm^2^, sum) of the area of interest using the Image Pro-Plus 7.0 software program (Media Cybernetics, Bethesda, MD).

### Statistical analysis

Statistical analysis was performed using the Prism 5.0 statistical package. A *T*-test was used to compare data between two groups. One-way ANOVA and the Bonferroni correction were used to compare data between three or more groups. Briefly, the data was first analyzed by ANOVA for mean differences among groups. When the ANOVA value was significant, Bonferroni’s multiple comparison tests were used to determine significance between specific groups. Values are expressed as mean values ± S.E.M. and a *p* value of <0.05 was considered statistically significant.

## Electronic supplementary material

Supplemental Figure Legend

Supplemental Figure 1

Supplemental Figure 2

Supplemental Figure 3

## Electronic supplementary material

Supplementary Information accompanies the paper on the *npj Precision Oncology* website (doi:10.1038/s41698-017-0011-4).
