# Supplementary material for: Inhibiting breast cancer by targeting the thromboxane A2 pathway
Source: NPJ Precis Oncol. 2017 Apr 3;1:8. doi: 10.1038/s41698-017-0011-4 (PMC5859468; doi:10.1038/s41698-017-0011-4)
Supplement: Supplementary file 1 — Supplemental Figure Legend [file 41698_2017_11_MOESM1_ESM.docx]

**Supplementary Figure Legends:**

**Supplementary Figure 1**. Knockdown of A, TBXAS1 or B, TBXA2R lowered the rate of cancer cell proliferation. Cell growth was evaluated by MTS assay. Mock and knockdown MCF-7 cells were seeded (1×10^3^ cells per well) in 96-well plates. After incubation for various times, 20 μL of CellTiter96^®^Aqueous One Solution (Promega) were added. Finally, the optical density was determined at 492 nm. Data are presented as mean values ± S.E.M. (n = 4). The asterisks indicate a significant difference compared with Mock group (***, *p* < 0.001).

**Supplementary Figure 2**. Effects of TXA_2_ modulators on TBXAS1 expression. A, effects of TXA_2_ modulators on TBXAS1 expression *in vitro*. 4T1 cells were treated with vehicle (DMSO), aspirin, indomethacin, celecoxib or ozagrel sodium for 48 h. Cell lysates were subjected to Western blot analysis. B, effects of indomethacin treatment on TBXAS1 expression *in vivo*. The mouse 4T1 breast tumor model was adopted for a breast cancer metastasis study. Indomethacin (1 mg/kg) or the vehicle (DMSO) was administered using an intragastric tube at 0.1 mL per 10 g every other day for a total of 28 days. Immunohistochemical staining of TBXAS1 expression was determined in the lung metastatic lesions. Data are presented as mean values ± S.E.M. (n = 5).

**Supplementary Figure 3**. Pathophysiological role of the PGE_2_ pathway in human breast cancer. Immunohistochemical staining of TBXAS1 or mPGES-1was performed in human breast cancer tissues or normal adjacent tissues. Original magnification: 40× and 200×.
