# Supplementary figures and images for: Inhibiting breast cancer by targeting the thromboxane A2 pathway
Source: NPJ Precis Oncol. 2017 Apr 3;1:8. doi: 10.1038/s41698-017-0011-4 (PMC5859468; doi:10.1038/s41698-017-0011-4)

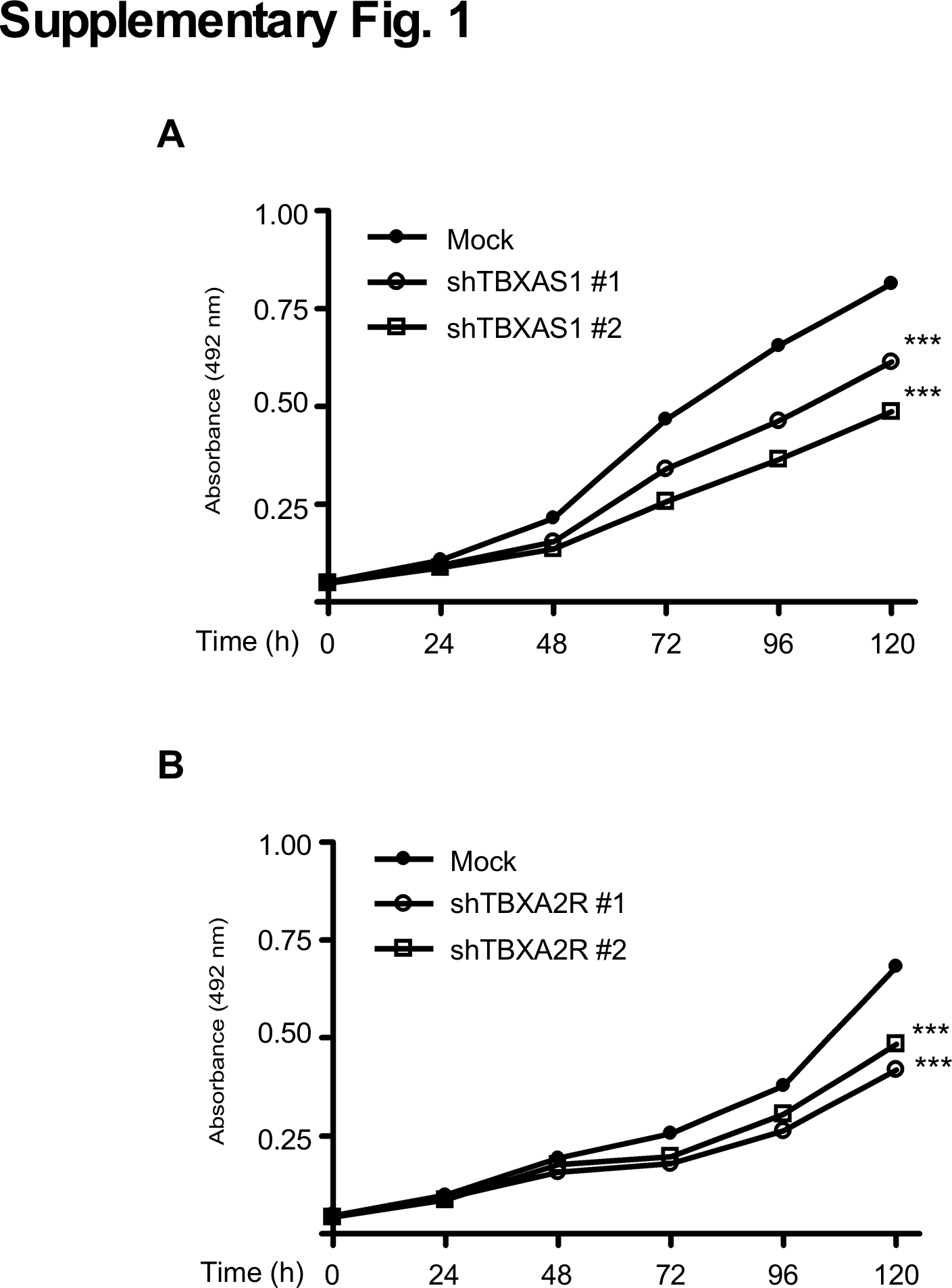

Supplement: Supplementary file 2 — Supplemental Figure 1 [file 41698_2017_11_MOESM2_ESM.tif]

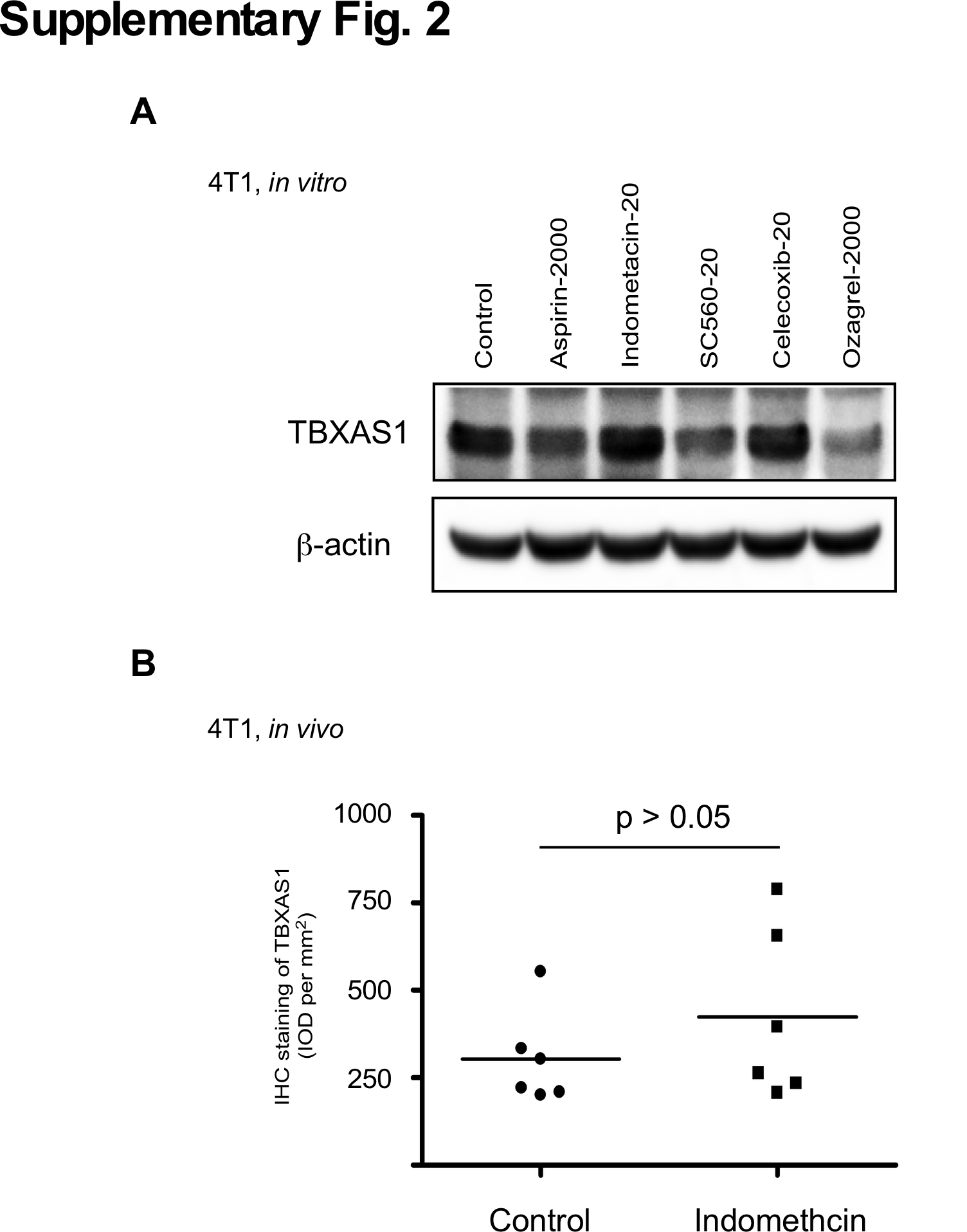

Supplement: Supplementary file 3 — Supplemental Figure 2 [file 41698_2017_11_MOESM3_ESM.tif]

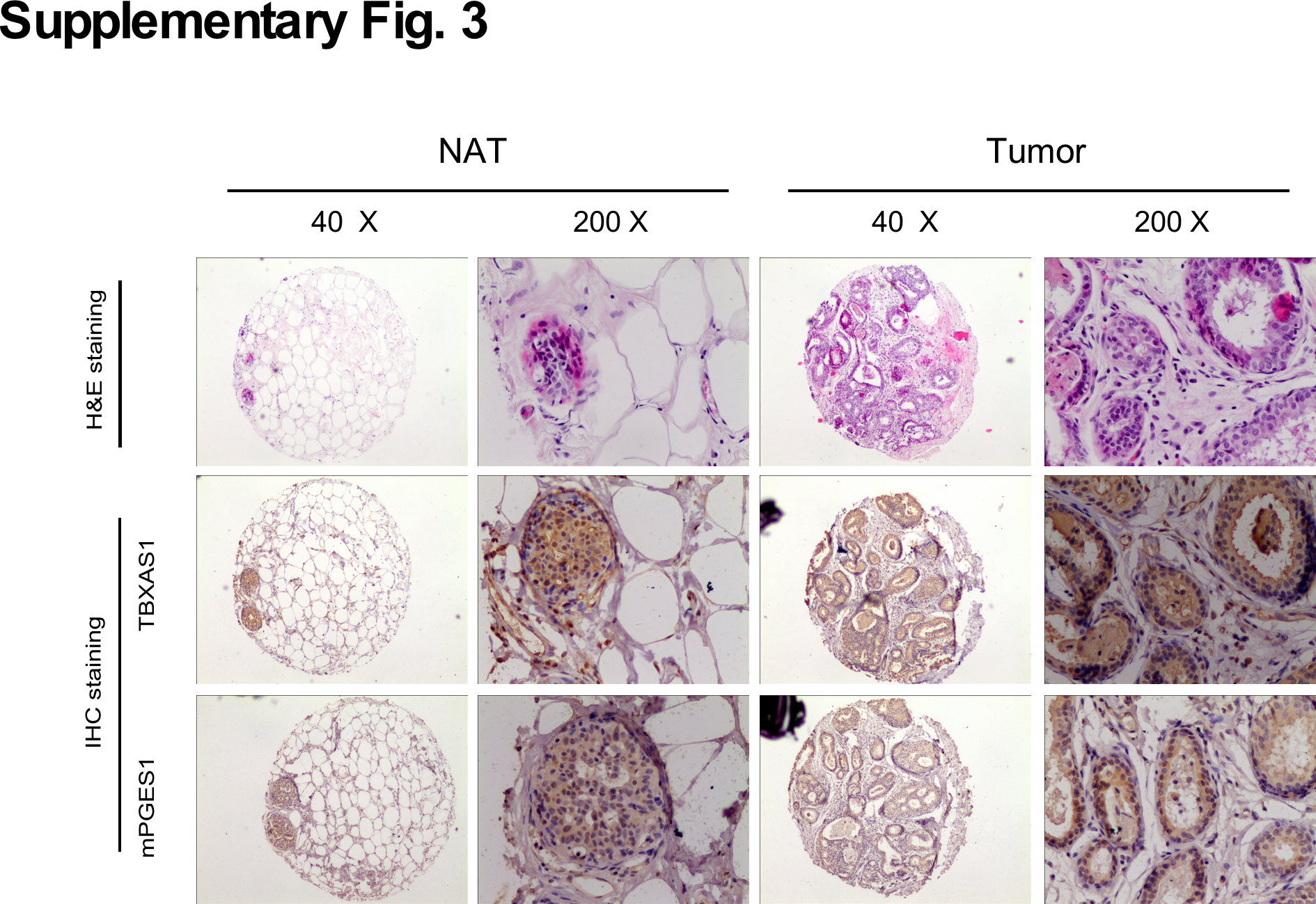

Supplement: Supplementary file 4 — Supplemental Figure 3 [file 41698_2017_11_MOESM4_ESM.tif]
